# The Impact of the Seasonal and Geographical Distribution of Tuberculosis in Sicily: A 6-Year Retrospective Study (2018–2023)

**DOI:** 10.3390/jcm13123546

**Published:** 2024-06-17

**Authors:** Ginevra Malta, Nicola Serra, Giovanni Francesco Spatola, Carmelo Massimo Maida, Giorgio Graziano, Domenico Di Raimondo, Teresa Maria Assunta Fasciana, Valentina Caputo, Anna Giammanco, Angela Capuano, Consolato M. Sergi, Antonio Cascio, Paola Di Carlo

**Affiliations:** 1 Department of Health Promotion, Mother and Child Care, Internal Medicine and Medical Specialties, University of Palermo, 90127 Palermo, Italy; ginevra.malta@unipa.it (G.M.); carmelo.maida@unipa.it (C.M.M.); domenico.diraimondo@unipa.it (D.D.R.); teresa.fasciana@unipa.it (T.M.A.F.); valentina.caputo@unipa.it (V.C.); antonio.cascio03@unipa.it (A.C.); paola.dicarlo@unipa.it (P.D.C.); 2 Department of Public Health, University Federico II of Naples, 80131 Naples, Italy; 3 Department of Biomedicine, Neurosciences and Advanced Diagnostics (BiND), University of Palermo, 90127 Palermo, Italy; 4 Clinical Epidemiology Unit, University Hospital “P. Giaccone”, 90127 Palermo, Italy; 5 School of Medicine and Surgery, University of Palermo, 90127 Palermo, Italy; 6 Department of Emergency, AORN Santobono-Pausilipon, 80122 Naples, Italy; 7 Anatomic Pathology Division, Pediatric Pathologist, University of Ottawa, Ottawa, ON K1H 8M5, Canada; csergi@cheo.on.ca; 8 Department of Laboratory Medicine and Pathology, University of Alberta, Edmonton, AB T6G 2R3, Canada

**Keywords:** tuberculosis, geographical area, seasonality, Sicily region

## Abstract

**Background:** Tuberculosis (TB) continues to be a major public health issue, with high mortality rates reported worldwide. It is worth noting that most of the hospitalizations for tuberculosis in the Sicilian region involve Italian-born individuals, underscoring the need to address this problem. Recent research on the geographic area and seasonality of infectious diseases, including tuberculosis, may aid in developing effective preventive measures. **Objectives**: This study aimed to evaluate the impact of the season and geographical area on tuberculosis disease prevalence in the Sicilian region. **Methods**: A retrospective study from January 2018 to May 2023 was conducted on patients with tuberculosis in the Sicilian region by analyzing computerized records on the Infectious Diseases Information System, currently named the Italian National Notification System (NSIS), of the Epidemiology Unit at Policlinico Paolo Giaccone University Hospital of Palermo and the Regional Reference Laboratory for Tuberculosis Surveillance and Control. **Results**: Eastern and Western Sicily were the geographical Sicilian areas with the highest frequency of patients with tuberculosis (52.2% and 42.6%, respectively). In comparison, Central Sicily had a significantly lower frequency of patients with tuberculosis (5.2%). Regarding the season, autumn was the season with the highest number of notification cases (28.9%), while spring was the season with the lowest frequency of patients with tuberculosis (19.7%). In autumn, we found significantly fewer patients with tuberculosis from Eastern Sicily (39.3%) and Central Sicily (1.5%), while Western Sicily had more patients with tuberculosis (59.3%). In spring, we found significantly more patients with tuberculosis from Eastern Sicily (64.1%), while Western and Central Sicily had significantly fewer patients with tuberculosis (23.9% and 12%, respectively). The presence of patients with tuberculosis did not significantly differ between geographical regions in summer and winter. **Conclusions**: Geographical area and seasonality significantly impact the distribution of tuberculosis cases in Sicily. These factors may be linked to different climatic conditions across the various geographical areas considered. Our findings suggest that climate can play a critical role in the spread of airborne infectious diseases, such as tuberculosis.

## 1. Introduction

Tuberculosis (TB) is a severe infectious disease caused by Mycobacterium tuberculosis (MTB). It primarily affects the lungs (pulmonary TB) but can also target other organs (extrapulmonary TB). TB is considered one of the leading causes of death worldwide. Since 1993, the World Health Organization (WHO) has been releasing yearly statistical reports about the incidence and prevalence of TB and the most effective prevention methods against infection. According to the latest WHO report in 2022, an estimated 10.6 million people have contracted tuberculosis (TB), and 1.6 million people have died because of the disease [1,2,3,4,5].

The WHO European region has developed a new action plan for the years 2023–2030 with the following targets for the year 2030 compared to the year 2015: reduce TB deaths by 90%, reduce the incidence of TB by 80%, and achieve a treatment success rate of 85% among cases of multi-drug-resistant TB (MDR-TB), especially in Eastern Europe [6,7,8,9,10].

The COVID-19 pandemic caused by Coronavirus-2 (SARS-CoV-2) has strongly impacted TB surveillance, prevention, and treatment activities [9,11,12].

Italy is defined by the WHO as a ‘low endemic’ country, as there are less than 10 cases of disease per 100,000 inhabitants. In 2021, 2480 cases of TB were reported. In 2021, the notification rate of TB was 4.2 cases per 100,000 residents; there was an increase of 8.4% compared to 2020 and a decrease of 25.9% compared to the values recorded in 2019. In Italy, TB is considered a class III disease and must be reported to the authorities. The Ministerial Decree of 15 December 1990 requires mandatory notification of TB cases, and there are guidelines for TB control that were updated in 1999 [13,14,15,16,17]. A surveillance sheet is used to monitor the disease. The local hospital or territorial healthcare workers enter the notifications into the Agency’s communication platform, and these notices are then directed to the Infectious Diseases Information System (SIMIWEB), currently named Italian National Notification System (NSIS). The Notification System of Infectious Diseases (PREMAL) of the Italian Higher Health Institute (Italian ISS) in the Sicilian Region has joined the SIMIWERB for TB notification [18].

In recent years, TB notification rates in Sicily have been gradually increasing. However, this region is still considered to be among the low-endemicity areas.

The season is connected to climate variables such as environmental humidity, temperature, atmospheric pressure, and wind, which can impact TB diffusion [19,20,21,22,23,24]. These events create an environment that facilitates the transmission and development of active TB, disrupting prevention and treatment services, especially in vulnerable countries [22,24].

Some studies, such as those about the impact of climate hazards sensitive to greenhouse gas on human pathogenic disease [25], have stressed the positive or negative effects of warming, precipitation and drought on human infectious diseases [26,27].

Limited Italian and Southern regional information is available about the climatic variability of TB and its geographical distribution, which makes decision-making challenging.

New interdisciplinary studies have emerged exploring the link between climate change and TB in a geographical area such as the Mediterranean. The sea plays a crucial role in the Mediterranean climate, but the recorded sea surface temperature (SST) anomalies impact the air temperature and precipitation in the surrounding areas [28].

### Objective

This study aims to evaluate the impact of the climate seasonally and by geographical area on tuberculosis frequency in the Sicily region.

## 2. Materials and Methods

We retrospectively analyzed notified TB cases in the Sicilian region from January 2018 to May 2023. All TB patients were identified using simple random sampling, and the data were collected by analyzing the computerized records of the Epidemiology Unit of Policlinico Paolo Giaccone University Hospital belonging to Infectious Diseases Information System (SIMIWEB), currently named the Italian National Notification System (NSIS), and the Notification System of Infectious Diseases (PREMAL) of the Italian Higher Health Institute, which are accessible from Policlinico “Paolo Giaccone” University Hospital Regional Reference Laboratory for Tuberculosis Surveillance and Control according to European Union Commission 2008/426/EC: Commission Decision of 28 April 2008 [29]. The PREMAL platform is a constantly updated interactive database that includes microbiologically confirmed TB cases, patients with symptoms or signs of TB, and/or images of TB disease.

Enrolled patients were all SARS-CoV-2-negative Italian-born patients resident in the Sicilian area with a first diagnosis of active pulmonary and extrapulmonary TB according to international guidelines and Regional Reference Laboratory for Tuberculosis Surveillance and Control at Policlinico Paolo Giaccone, University of Palermo Italy [30,31,32].

Ethical committee approval and patients’ informed consent were not required, because the data were anonymously obtained by a computerized system, according to the Italian Data Protection Authority. Regional health authorities routinely use anonymous data for epidemiological and administrative purposes.

### 2.1. Geographical Sicilian Area

**Figure 1:**
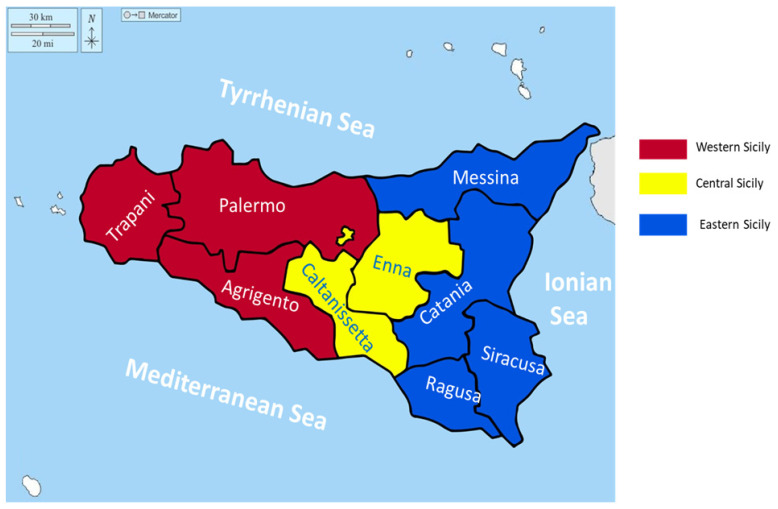
Geographical areas of the Sicily region.

Sicily is an island in Southern Italy with a surface of 25.711 km^2^ (longitude: 14.0153557, latitude: 37.5999938, mean elevation: 622 m/2041 feet, and barometric pressure: 94 Kpa). The island is predominantly mountainous, characterized by Mount Etna (10,900 feet, 3220 m), with intense volcanic activity. The region of Sicily is divided into nine provinces: Palermo (PA), Trapani (TP) and Agrigento (AG) in the west; Caltanissetta (CL) and Enna (EN) in the center; and Ragusa (RG), Siracusa (SR), Catania (CT) and Messina (ME) in the east (Figure 1) [33]. The post-census population in 31 December 2022 in Sicily was 4,814,016 residents, distributed as follows between the three geographical areas: west = 2,032,372, center = 404,371, and east = 2,377,273 [33].

### 2.2. Seasonal and Geographical Area Variables

Since our study aim was to evaluate the impact of climate on TB disease in Sicily, with the aim of adequate prevention, we considered two basis variables in this study: seasonality and geographical area. Environmental parameters such as temperature, atmospheric pressure, precipitation index, etc., were not considered individually, as they are parameters that do not have standard variations but are highly variable. Therefore, the use of these parameters could bring a bias to our statistical analyses. The authors overcame this bias by considering a standard measure of climate variations in Italy; this measure is represented by the seasons. This can be considered a macro parameter that involves all the others. Mathematically, this step is the basis of reducing statistical biases, i.e., identifying a standard and dominant parameter that can include all the other sub-parameters with greater variability.

A similar consideration is made for the geographical area in which reference is not made to the single city of Sicily, but to an entire area. Also, in this case, the fluctuations are reduced to obtain a more reliable overview of the situation in Sicily, and to be more effective in prevention.

### 2.3. Statistical Analysis

Data are presented as number and percentage for categorical variables, and continuous data are expressed as mean ± standard deviation (SD), and median and interquartile interval (IRQ = [Q1; Q3]).

The test for a normal distribution was performed using the Shapiro–Wilk test. The t-test was used to test the differences between two means of unpaired data. Alternatively, the Mann–Whitney test was used if the distributions were not normal. Particularly, where the tests on medians showed a significant difference and the medians were equal, the mean rank values were described. The Kruskal–Wallis test, followed by a post hoc test with Conover test for pairwise comparison, was performed in multi-comparison among three or more independent random samples in the case of no normal distribution condition. Particularly, in the case of groups with distributions with different shape, the analyses were performed on mean ranks. Multiple-comparison chi-square test was used to define significant differences among three or more independent variables. If the chi-square test was significant (*p* < 0.05), a post hoc Z-test was performed to identify the highest or lowest significant frequency. Particularly, Fisher’s exact test was used where the chi-square test was not appropriate. In addition, the chi-square goodness of fit was used to evaluate significant differences among three or more variable modalities. The binomial test was performed to compare two mutually exclusive proportions or percentages. All data were analyzed using the MATLAB statistical toolbox version 2008 (MathWorks, Natick, MA, USA) for 32-bit Windows.

## 3. Results

A total of 467 Italian patients with TB were enrolled between 2018 and 2023 in the Sicily region. In Table 1, we report the general characteristics of our sample such as gender and age at diagnosis, including Sicilian geographical area and seasonality.

From Table 1, we observed a more significant presence of males than females with TB in the Sicilian region (65.3% vs. 34.7%, *p* < 0.0001).

In Table 2, we investigate the impact of season on patients with TB, particularly the possible relationships between season and gender, age at admission and geographical Sicilian area.

In Table 2, in the last column, we report the statistical analysis across seasons, i.e., across columns. We found significant differences regarding the presence of patients with TB across seasons (*p* = 0.0378) and across Sicily’s geographical areas (*p* < 0.0001). Particularly, we found a significantly higher frequency of patients with TB in autumn (28.9%, *p* = 0.0449) and a significantly lower presence in spring (19.7%, *p* = 0.0105).

In autumn, we found a significantly lower presence of patients with TB from Eastern Sicily (39.3%, *p* = 0.0003) and Central Sicily (1.5%, *p* = 0.0221), while from Western Sicily, a significantly greater presence of patients with TB was observed (59.3%, *p* < 0.0001). In spring, we found a significantly greater presence of patients with TB from Eastern Sicily (64.1%, *p* = 0.0116), while from Western and Central Sicily, a significantly lower presence of patients with TB was observed (23.9%, *p* = 0.0001;12%, *p* = 0.0010).

No significant presence of TB patients across different Sicily areas was observed in summer and winter. Finally, no significant differences in patients with TB across different seasons for gender, age at admission, age at admission for males and age at admission for females were observed (*p* = 0.19; *p* = 0.10, *p* = 0.28, *p* = 0.17, respectively).

Additional analyses were performed considering each season, i.e., for each column and considering variables such as gender and geographical area. We found for gender a significant presence of males in summer (70.7%, *p* < 0.0001), autumn (63.0%, *p* = 0.0025) and winter (68.4%, *p* = 0.0001), while for spring, no significant differences for gender were observed (M: 57.6% vs. F: 42.4%, *p* = 0.14).

In summer, Eastern and Western Sicily were the geographical area with a greater presence of patients with TB (52%, *p* < 0.0001; 42.3%, *p* = 0.0232; respectively), while Central Sicily was the area with a lower frequency of patients with TB (5.7%, *p* < 0.0001).

In autumn, Western Sicily was the geographical area with a greater presence of patients with TB (59.3%, *p* < 0.0001), while Central Sicily was the area with a lower frequency of patients with TB (1.5%, *p* < 0.0001).

In winter, Eastern Sicily was the geographical area with a greater presence of patients with TB (58.1%, *p* < 0.0001), while Central Sicily was the area with a lower frequency of patients with TB (3.4%, *p* < 0.0001).

Finally, in spring, Eastern Sicily was the geographical area with a greater presence of patients with TB (64.1%, *p* < 0.0001), while Central Sicily was the area with a lower frequency of patients with TB (12%, *p* < 0.0001).

We observed that the geographical areas with more TB patients were subjects from Eastern Sicily and Western Sicily.

In Table 3, we investigate the impact of geographical Sicilian area on patients with TB, particularly the possible relationships between geographical area and gender, age at admission and seasonality.

In Table 3, in the last column, we report a statistical analysis across Sicilian geographical areas, i.e., across columns. We found that a greater presence of patients with TB were from Eastern Sicily (52.2%, *p* < 0.0001) and Western Sicily (42.6%, *p* < 0.0001), while in Central Sicily, we found a significantly lower presence of patients with TB (5.2%, *p* < 0.0001). Notably, we checked that these results were not affected by biases linked to the population distribution in the different Sicilian areas. For this scope, we considered the population of Sicily residing in the three macro areas considered, as reported in the Section 2. We compared the ratios of the number of residents between different areas and the ratio of the number of correspondent cases with active TB identified in this study. In this step, we obtained that the ratios between Western and Central, and Eastern and Central Sicily were less than those of TB cases (5.02 vs. 8.29, *p* = 0.0192; 5.88 vs. 10.17, *p* = 0.0095). In this case, the ratios were significantly different, i.e., they were not conditioned by population distribution. We reported in detail this analysis:

Western/Eastern = 0.85 (2.032.372/2.377.273), TB = 0.82 (199/244, chi-square = 0.24, *p* = 0.62),

Western/Center = 5.02 (2.032.372/40.4371), TB = 8.29 (199/24), chi-square = 5.48, *p* = 0.0192),

Eastern/Center = 5.88 (2.377.273/40.4371), TB = 10.17 (244/24), chi-square = 6.73, *p* = 0.0095).

In addition, considering gender by age at admission, we found that males with TB had a significantly lower age in Central Sicily compared to Eastern and Western Sicily (median: 25 < 55, *p* < 0.05; 25 < 60, respectively).

A significant relationship was found between geographical area in Sicily and seasonal variation (Table 2).

No significant differences in patients with TB between geographical area and gender and between geographical area, age at admission, and age at admission for females were observed (*p* = 0.93; *p* = 0.18, *p* = 0.07, respectively).

Furthermore, we analyzed geographical area, i.e., for each column, considering variables such as gender and seasonality.

We found for gender a significant presence of males for Eastern and Western Sicily (66%, *p* < 0.0001; 64.8%, *p* < 0.0001), while for Central Sicily, no significant differences regarding gender were observed (*p* = 0.22).

In Eastern and Central Sicily, we observed no significant differences for patients with TB across seasons (*p* = 0.56, *p* = 0.053, respectively), while we found in Western Sicily a greater presence of patients with TB in autumn (40.2%, *p* < 0.0001) and a smaller presence of patients in spring (11.2%, *p* < 0.0001).

**Figure 2:**
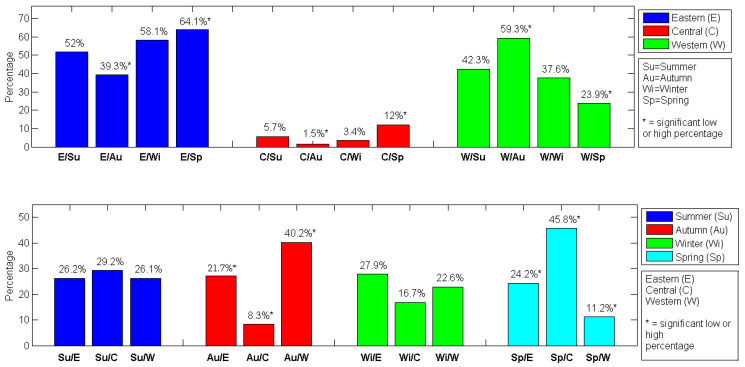
Percentages of TB patients grouped by geographical area for each season (**a**) and by season for each geographical area (**b**). The asterisks represent low or high significant percentages.

In Figure 2, we show graphically the TB patients grouped by geographical area for each season (Figure 2a) and by season for each geographical area (Figure 2b), according to the last row of Table 2 and Table 3, respectively.

**Figure 3:**
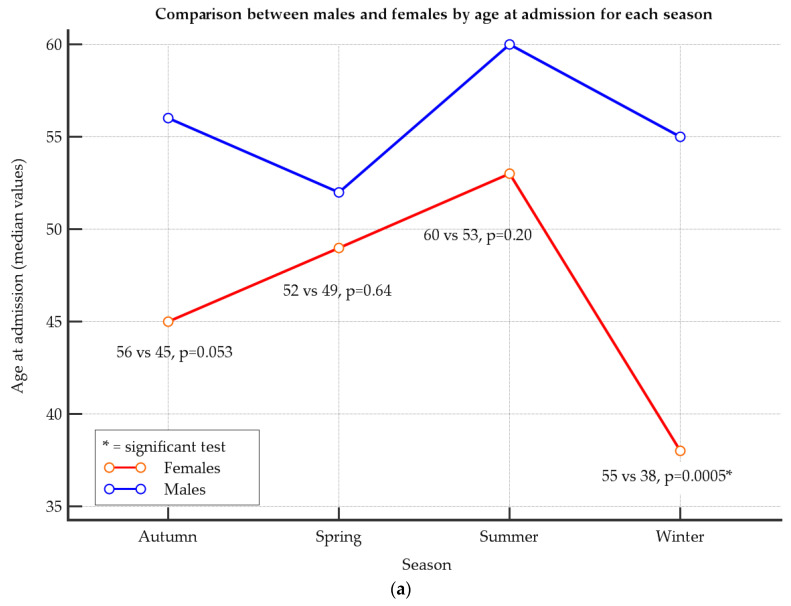

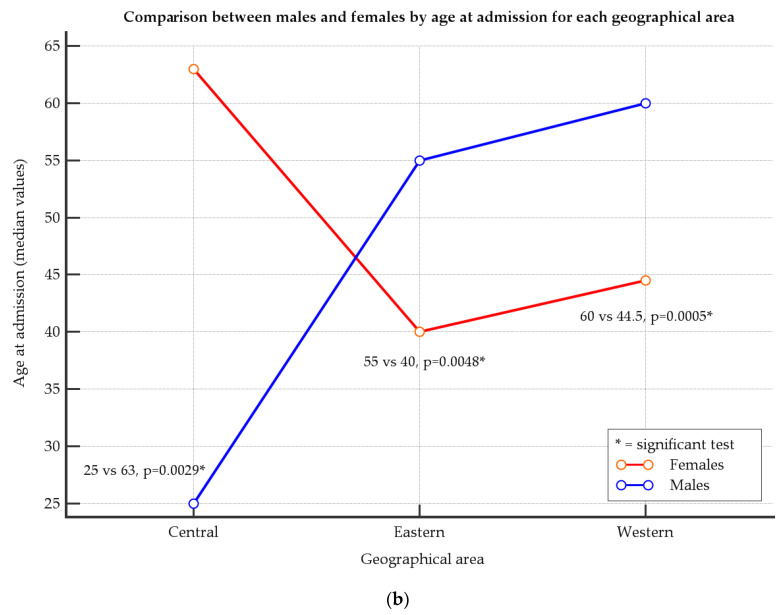
Comparison between males and females by age at admission for each season (**a**) and geographical area (**b**).

Finally, in Figure 3, we report the distribution of age at admission by gender for each season (Figure 3a) and for each geographical area (Figure 3b), according to rows 4 and 5 of Table 2 and Table 3, respectively. Particularly, comparisons between males and females were reported regarding age at admission for each season (Figure 3a) and geographical area (Figure 3b).

Figure 3 shows a significantly higher age for males than females with active TB in winter (median: 55 vs. 38, *p* = 0.0005) and in Western and Eastern areas (median: 60 vs. 44.5, *p* = 0.0005; 55 vs. 40, *p* = 0.0048, respectively). Vice versa, in Central Sicily, a significantly higher age for females than males with active TB was observed (median: 63 vs. 25, *p* = 0.0029).

## 4. Discussion

Italy is considered a low-burden country for TB. The number of TB cases reported in the country remained relatively stable between 2013 and 2018. Among the years considered, the lowest number of TB cases was reported in 2020, with a total of 2287 cases. The highest number of cases was recorded in 2021, with a total of 2378 cases, corresponding to an incidence rate of 4.9 per 100,000 and 4.13 per 100,000 people in 2022 [34,35]. On the other hand, the lowest number of TB cases reported was in 2016, with a total of 4032 cases. Northern and Central Italy regions have higher TB notification rates (7.9 and 7.8 per 100,000, respectively) than Southern Italy and the islands (3.1 and 3.6 per 100,000, respectively from 2009 to 2021) [34]. Pipitò et al. reported that in Sicily, 3745 people were hospitalized for TB, with 5239 admissions and 166 deaths from 2009 to 2021 [36].

The data showed no significant differences in patients with TB across geographical areas, while for each geographical area, we found a predominantly male gender (Eastern: M = 66% vs. F = 34%; Central: M = 62.5% vs. F = 37.5%; Western: M = 64.2% vs. F = 35.2%), as observed worldwide for this disease [37,38].

Regarding the ages of males and females, there was no significant age difference across seasons (Table 2). At the same time, across geographical areas, we found a higher age in males with active TB in Eastern and Western than in Central Sicily (Table 3). Regarding the comparison between males and females, we did not find any significant differences for each season except in winter, where a significantly higher age in male than female patients was observed. The significantly higher occurrence of TB in older men compared to women during winter may be attributed to unhealthy lifestyles, such as smoking. Research has shown that smoking increases the risk of contracting TB [39]. In Southern Italy, men tend to consume more cigarettes as they age compared to women [40]. It is hypothesized that cigarette consumption may increase during winter when people stay at home and indoors [41,42]. In addition, for each geographical area, we found a significantly higher age in males than females in the Western and Eastern area than Central Sicily, where a significantly younger age for male gender was observed (Figure 3).

The age of active TB notification by gender in Central Sicily is opposite in coastal areas (Western and Eastern Sicily). The concordance of the results obtained for coastal zones may be linked to greater access of males to the health care service for screening for health status. In fact, in humans, there are greater risk factors predisposing them to TB, and a greater percentage of employment levels in work contexts are associated with a greater risk of respiratory diseases [43].

Regarding the female gender, it has been observed that hormonal fluctuations during the menstrual cycle can cause changes in the number and function of immune cells. This can worsen certain diseases, including susceptibility to TB. Moreover, the higher prevalence of TB disease, previously attributed to sociocultural inhibitions in women regarding expectoration, may have a hormonal reason. Estrogen reduces the liquid layer of the airway epithelium, while progesterone decreases the beat of cilia, as reported by Rossitto S. et al. [44].

Identifying geographical areas and seasons with greater diffusion of infectious airborne diseases such as TB could help define adequate social and health measures to reduce the risk of TB transmission. [45,46,47]. In our study, we found more frequent cases of TB in Eastern and Western Sicily than in the Central area (Table 1 and Table 3). The distribution pattern of TB may potentially depend on the degree of urbanization in different regions of a Sicilian provinces. Notably, the Eastern and Western Sicilian areas may experience a higher incidence of TB due to a greater presence of coastal cities with high population density than the Central Sicilian zone, a rural area with a low population density. Our results are in accordance with prior studies that reported that urbanization can increase the risk of TB transmission compared to rural areas with low population density [45,46]. Further exploration of additional variables, such as sociodemographic factors, geographical locations, and seasonality, may help our comprehension of the relationship between these factors and the distribution of TB cases [47,48,49,50].

This status shows that in the diagnostic approach to date, there are shortcomings in the investigation of risk factors, where the subject’s area of origin should be included, and not just the possible presence of trips abroad; this is further affected by the daily debated impact of climate change on human health, which no longer forces the spread of certain infectious diseases, including TB, to be limited to historic endemic areas [51].

In our study, seasonality influenced TB notification cases. Autumn was the season with more TB patients (28.9%) than others (Table 2). This result could be a consequence of previous infection during the spring and summer months characterized by overcrowding due to higher social activities with high human social interaction. Previous studies have shown that contact outside the household, e.g., social drinking may be important for the transmission of TB [52,53]. Moreover, schools and universities in Italy start their didactic work, activities, initiatives, and projects in autumn. “Summer fun” could be responsible for TB infection, which advances from infection to symptomatic diseases and to diagnosis in the following months. This statement could support the findings of our study, which showed a higher incidence of TB cases during the autumn season. Furthermore, in our study, we found a higher number of cases of TB reported in the Eastern (52.2%) and Western (42.6%) Sicilian area (Table 3). These areas include all coastal, more urbanized cities with a high transit of people such as Palermo, Catania, Trapani, Agrigento, and Messina.

In addition, we found a relationship between geographical areas and seasonality. Particularly, more TB cases were observed in autumn in the Western zone (59.3%) and in the spring season in both the Eastern (61.1%) and Central (12%) Sicilian areas (Table 2). These findings can be due to better connection due to short travel distances and a better interurban public transport service between the Eastern and Central areas.

It has been observed that the occurrence of TB varies seasonally in several populations and countries with different incidence levels. This phenomenon has been reported since as early as the 1930s, and has recently been highlighted in European and other countries, showing the seasonal variation of TB disease [54,55,56,57,58].

Respiratory illnesses, such as influenza, have been found to exhibit a seasonal pattern, with a higher incidence occurring during the winter months [59]. This phenomenon has been widely observed and attributed to various factors, including changes in humidity, temperature, and human behavior. The exact mechanisms driving seasonal fluctuations in respiratory disease incidence remain unknown, but some research suggests that environmental conditions and host susceptibility play a significant role [22,23,24,25,58]. Therefore, understanding the seasonal patterns of respiratory infections is crucial for public health organizations to develop effective preventive measures and treatment strategies [55,57].

Notably, the factors responsible for the seasonality of TB exhibit a greater complexity than those that drive most respiratory illnesses. While the incidence of the latter would promptly respond to any seasonal drivers, the nature and timing of TB’s underlying forces are far more intricate in the different stages of disease evolution.

Investigating the correlation between the incidence of TB notification cases and the season has yielded insightful results [54,55,56,57,58,60]. Our findings indicate a higher prevalence of TB diagnoses during the months of autumn. This result is partially in accordance with Manabe T. et al. [60], who described a monthly trend of Japanese TB notification cases more frequent from spring to autumn. Particularly, in our study, considering the single Sicilian area, we found more frequent notification cases of TB in spring for Eastern (24.2%) and Central Sicily (45.8%) than Western Sicily (11.2%), as reported in Table 3. This could be caused by the better interurban connection between Central and Eastern Sicilian areas, which favors the number of people travelling from Central to Eastern areas in the direction of coastal cities in spring when the temperature becomes favorable. The pleasant temperature in Sicily in spring determines an increase in the number of tourists [61], and overcrowding and increased contact, and consequently an increase in airborne transmission infections such as TB. Vice versa, in autumn, we found in Western Sicily more notification cases of TB (40.2%) than in Eastern (21.7%) and Central Sicily (8.3%). This result is probably due to daily travel linked to occupation and both school and university activities [62]. In addition, these findings indicate that the greatest number of cases are typically reported during the transitional seasons of spring and autumn. TB resurgence may be due to its prolonged incubation period and potential for reactivation after the initial infection. This reactivation could occur during any season, including summer or winter, especially among individuals living in restricted communities or disadvantaged socio-economic conditions, as recent observations have shown [60,63]. Of course, a higher rate of people with TB in each area could be linked to the type of climate in that area. The simultaneous occurrence of wildfires, like the ones seen in Southern Italy, especially in the Sicily region, and increased air pollution linked to season can cause respiratory complications such as coughing and respiratory diseases. This can lead to the spread of airborne diseases like TB in some geographical areas [64,65]. Furthermore, such climatic events can profoundly impact on the health of populations in affected areas, requiring timely measures to mitigate their negative consequences [63,65,66]. At the basis of the phenomenon facilitating the spread of TB related to climate change is also the same biological basis of Mycobacterium tuberculosis. The metabolic functions of mycobacteria are reduced at colder temperatures and higher altitudes. Their ability to replicate and survive is inhibited [20,66]. Finally, radon gas and its byproducts have been extensively studied on Mount Etna, including their correlation with volcanic activity and magma dynamics. Moreover, in Eastern Sicily, seasonal rainfall-triggered hydro-mechanical processes and their feedback with deep magmatic activity require further investigation in the context of climate change. Due to the potential danger to human health, public health authorities and the scientific community should pay special attention to this topic. Respiratory diseases have been linked to human exposure to volcanic emissions, along with TB. Therefore, infectious respiratory diseases could be investigated along with volcano activity in Catania province, Eastern Sicily, and seasonality [67,68].

## 5. Conclusions

The study results have brought to light that the incidence of TB is higher in the coastal and urbanized regions of Eastern and Western Sicily, compared to the central areas where the notification case was higher in spring. This result suggests that the disease is linked to environmental factors prevalent in the region’s densely populated and urbanized areas. An aspect of TB prevention is the geographical and seasonal factors that could influence the prevalence rate of TB in the Sicilian region. Consequently, determining the areas with a greater prevalence of TB can be instrumental in implementing necessary preventive measures. Identifying areas within Sicily with a higher population density of individuals affected by TB is critical in its prevention. It is essential to consider specific geographical and seasonal factors that may influence the prevalence rate of the disease in this region. This study underscores the importance of further investigation into these factors, which could impact public health policies and interventions that are aimed at reducing the spread of the disease.

## 6. Limitations

The focus of this study was to evaluate the trend of active TB cases based on seasonality. We have not included cases of TB in migrants because the migratory phenomenon is uncontrollable; in fact, migrants are often illegal immigrants or in transit for variable periods in different Sicilian territories or other regions, and therefore, the monitoring and identification of migrants with active TB is very random and could introduce a statistical bias in our analyses.

TB is a disease that spreads when a person inhales droplets containing *M. tuberculosis* into their lungs. Regardless of the type of TB, it can be transmitted from person to person. Therefore, this study did not consider TB forms and socioeconomic factors, which is the only descriptive limitation of our study performed in a high-income country. The main objective of this study was to identify the predictive factors that affect the distribution of active TB cases linked to climate, such as the geographical area and seasonality, to strengthen and improve prevention measures. In this context, knowledge of the type of TB and socioeconomic factors does not impact this cross-sectional observational study.

## Figures and Tables

**Table 1:** General characteristics of total patients.

Parameters	% (n)
Italian patients with active TB	467
Age at admission	
Mean (SD)	Total: 49.5 (22.7); M:52.5 (21.7); F: 44.0 (23.6)
Median (IQR)	Total: 52 (31.25–69); M: 55 (37–71); F:44 (25–63)
Gender	
Male	65.3% (305)
Female	34.7% (162)
Sicilian geographical area	
Eastern Sicily *	52.2% (244)
Western Sicily #	42.6% (199)
Central Sicily §	5.2% (24)
Season	
Autumn	28.9% (135)
Summer	26.3% (123)
Winter	25.1% (117)
Spring	19.7% (92)

M = males, F = females, * Ragusa (RG), Siracusa (SR), Catania (CT) and Messina (ME) cities and province in the Eastern area; # Palermo (PA), Trapani (TP) and Agrigento (AG) cities and province in the Western area; § Caltanissetta (CL) and Enna (EN) cities and province in the Central area.

**Table 2:** Relationship between seasonality and age, gender, and geographical area in Sicilian patients with TB.

	Summer (Su) % (n)	Autumn (Au) % (n)	Winter (Wi) % (n)	Spring (Sp) % (n)	Analysis across Seasons *p*-Value (Test)
Patients (n = 467)	26.3%	28.9%	25.1%	19.7%	*p* = 0.0378 * (Cgf)
	(123)	(135)	(117)	(92)	Au: 28.9% **, *p* = 0.0449 (Z)
					Sp: 19.7% ***, *p* = 0.0105 (Z)
Age at admission					
Mean (SD)	53.7(20.1)	49.7(23.4)	47.1(23.1)	46.8 (24.0)	
Median (IQR)	57 (40–71)	52(28.5–70.75)	50 (27–66.5)	50(25–66)	*p* = 0.10 (KW)
Age at admission for males					
Mean (SD)	55.4 (19.3)	52.7 (23.2)	52.1 (21.4)	47.8 (23.2)	
Median (IQR)	60.0 (41.5–71.8)	56.0 (36.5–74.0)	55.0 (39.0–68.5)	52.0 (27.3–65.3)	*p* = 0.28 (KW)
Age at admission for females					
Mean (SD)	49.7 (21.7)	44.7 (23.1)	36.2 (23.3)	45.4 (25.2)	
Median (IQR)	53.0 (30.5–67.5)	45.0 (26.0–63.0)	38.0 (16.0–54.0)	49.0 (25.0–70.8)	*p* = 0.17 (KW)
Gender					*p* = 0.19 (C)
Male	70.7% (87) **	63.0% (85) **	68.4% (80) **	57.6% (53)	
Female	29.3% (36)	37.0% (50)	31.6% (37)	42.4% (39)	
					*p* < 0.0001 * (F)
Geographical area					
Eastern Sicily	52.0% (64) **	39.3% (53)	58.1% (68) **	64.1% (59) **	Au–Western: 59.3% **, *p* < 0.0001 (Z)
Central Sicily	5.7% (7) ***	1.5% (2) ***	3.4% (4) ***	12.0% (11) ***	Sp–Western: 23.9% ***, *p* = 0.0001 (Z)
Western Sicily	42.3% (52) **	59.3% (80) **	37.6% (44)	23.9% (22)	Sp–Eastern: 64.1% **, *p* = 0.0116 (Z)
					Au–Eastern: 39.3% ***, *p* = 0.0003 (Z)
					Au–Central: 1.5% ***, *p* = 0.0221 (Z)
					Sp–Central: 12% **, *p* = 0.0010 (Z)

* = significance test; ** = modality significantly more frequent; *** = modality significantly less frequent; C = chi-square test; KW = Kruskal–Wallis test; Cgf = chi-square goodness of fit; Z = post hoc Z-test F = Fisher’s exact test. The percentages are reported in each column; in the last column, statistical tests were performed across columns.

**Table 3:** Relationship between geographical area and, age, gender and season in Sicilian patients with TB.

	Eastern Sicily % (n)	Central Sicily % (n)	Western Sicily % (n)	Analysis across Sicilian Areas *p*-Value (Test)
				*p* < 0.0001 * (Cgf)
Patients (n = 467)	52.2% (244) **	5.2% (24) ***	42.6% (199) **	Eastern: 52.2% **, *p* < 0.0001 (Z)
				Central: 5.2% ***, *p* < 0.0001 (Z)
				Western: 42.6% **, *p* < 0.0001 (Z)
Age at admission				
Mean (SD)	48.5(23)	43.8 (22.6)	51.5 (22.4)	
Median (IQR)	52 (28–67.5)	41.5 (21.5–63)	53 (37–71)	*p* = 0.18 (KW)
Age at admission for males				*p* = 0.0009 * (KW)
Mean (SD)	51.8 (21.2)	33.3 (19.2)	55.6 (21.5)	Central < Eastern, *p* < 0.05 (Co)
Median (IQR)	55.0 (38.0–69.0)	25 (19–49)	60.0 (41.8–73.3)	Central < Western, *p* < 0.05 (Co)
Age at admission for females				
Mean (SD)	42.3 (24.9)	61.2 (16.3)	43.9 (22.2)	
Median (IQR)	40.0 (20.0–65.5)	63 (52.8–71.5)	44.5 (26.0–60.0)	*p* = 0.07 (KW)
Gender				*p* = 0.93 I
Male	66.0% (161) **	62.5% (15)	64.8% (129) **	
Female	34.0% (83)	37.5% (9)	35.2% (70)	
				*p* < 0.0001 * (F)
Season				
Summer (Su)	26.2% (64)	29.2% (7)	26.1% (52)	Eastern–Sp: 24.2% **, *p* = 0.0109 (Z)
Autumn (Au)	21.7% (53)	8.3% (2)	40.2% (80) **	Eastern–Au: 21.7% ***, *p* = 0.0003 (Z)
Winter (Wi)	27.9% (68)	16.7% (4)	22.6% (45)	Central–Au: 8.3% ***, *p* = 0.0224 (Z)
Spring (Sp)	24.2% (59)	45.8% (11)	11.2% (22) ***	Central–Sp: 45.8% **, *p* = 0.0010 (Z)
				Western–Au: 40.2% **, *p* < 0.0001 (Z)
				Western–Sp: 11.2% ***, *p* = 0.0001 (Z)

* = significance test; ** = modality significantly more frequent; *** = modality significantly less frequent; C = chi-square test; KW = Kruskal–Wallis test; Cgf = chi-square goodness of fit; Z = post hoc Z-test; F = Fisher’s exact test; the percentages are reported in each column. In the last column, statistical tests were performed across columns.

## Data Availability

Data are accessible only with the authorization of Epidemiology Unit of Policlinico Paolo Giaccone University Hospital at Infectious Diseases Information System (SIMIWEB, currently named Italian National Notification System (NSIS), available at https://sso.nsis.sanita.it/) and the Notification System of Infectious Diseases (PREMAL) of the Italian Higher Health Institute, available at https://www.salute.gov.it/portale/malattieInfettive/dettaglioContenutiMalattieInfettive.jsp?lingua=italiano&id=650&area=Malattie%20infettive&menu=sorveglianza (accessed on 10 June 2024).
